# Total Neoadjuvant Therapy for Locally Advanced Rectal Cancer: How to Select the Most Suitable?

**DOI:** 10.3390/jcm13175061

**Published:** 2024-08-26

**Authors:** Chanyoot Bandidwattanawong

**Affiliations:** Division of Medical Oncology, Department of Internal Medicine, Faculty of Medicine, Navamindradhiraj University, Bangkok 10300, Thailand; chanyootmd@gmail.com

**Keywords:** locally advanced rectal cancer, total neoadjuvant therapy, rectal cancer, customized paradigm of management

## Abstract

Rectal cancer shows specific characteristics in terms of pattern of recurrence, which occurs commonly at both local and distant sites. The standard of care for locally advanced rectal cancer (LARC) including neoadjuvant chemoradiotherapy, followed by surgery based on the total mesorectal excision principles leads to a reduction in the rates of local recurrences to 6–7% at 5 years. However, the outcomes among those with high-risk lesions remain unsatisfactory. On the contrary, neoadjuvant chemoradiotherapy results in long-term morbidities among those with low-risk lesions. Furthermore, the overall survival benefit of neoadjuvant therapy is still a subject to be debated, except for patients with complete or near-complete response to neoadjuvant therapy. Total neoadjuvant therapy (TNT) is a new paradigm of management of high-risk rectal cancer that includes early administration of the most effective systemic therapy either before or after neoadjuvant radiotherapy with or without chemotherapy prior to surgery with or without adjuvant chemotherapy. TNT potentially improves disease-free survival, even though whether it can prolong survival has been debatable. Recently, neoadjuvant chemotherapy only has been proved to be non-inferior to neoadjuvant chemoradiotherapy in patients with low-risk lesions. This review intends to review the current evidences of neoadjuvant therapy and propose a more customized paradigm of management of LARC.

## 1. Introduction

Colorectal adenocarcinoma (CRC) is the worldwide third most commonly diagnosed cancer. Approximately one-third of cases occur in the rectum [1]. Rectal cancer, specifically defined as the distal part of intestine traversing the pelvic cavity and below the peritoneal reflection, has many distinct features that merit attention. The propensity in loco-regional recurrence within the pelvic cavity in rectal cancer is higher than cancer arising within the rest of the colon due to its close proximity of the rectum to pelvic structures, absence of a serosa enveloping the rectum and surgical difficulties associated with obtaining both wide circumferential and long vertical surgical resection margins. Moreover, the lower rectum has unique lymphatic drainage. Lymphatic spreading from low-lying tumor tends to pass laterally to lymph nodes along the pelvic side wall, so the called lateral pelvic lymph nodes. This further makes surgical technique more challenging. Owing to these characteristics, locoregional therapy, especially radiotherapy (RT) with or without concomitant chemotherapy is often applied as a neoadjuvant or an adjuvant therapy in the curative management of stage II and III rectal cancer [2]. With the advent of total mesorectal excision (TME), the cumulative incidence of local recurrence of 6% at 5 years and 8% at 10 years was reported based on the data by Heald et al., significantly less than 10% or more compared with historical reports [3]. When it was further incorporated with RT, there were even fewer locoregional recurrences. The Dutch TME trial demonstrated that the 5-year local recurrence rate significantly decreased from 10.9% among patients undergoing TME alone to 5.6% among patients undergoing TME with pre-operative short-course radiotherapy (SC-RT) (*p* < 0.001). Even though the overall survival (OS) was not different (63.5% and 64.2%, respectively; *p* = 0.902), subgroup analyses revealed that the effect in reducing risk of local recurrence was limited to patients with nodal involvement, with lesions between 5 and 10 cm from the anal verge (AV), and patients with uninvolved circumferential resection margins (CRM) [4]. Since the German trial (CAO/ARO/AIO-94) by Sauer et al. was reported, the pre-operative (neoadjuvant) long-course chemoradiotherapy (LC-CRT) has been the standard of care for locally advanced rectal cancer (LARC) due to its superiority over post-operative CRT. The 10-year cumulative incidence of local relapses among patients receiving pre-operative LC-CRT remained lower than those receiving post-operative (7.1% vs. 10.1%; *p* = 0.048). However, neither the 10-year cumulative incidence of distant metastases (DM) (29.8% vs. 29.6%; *p* = 0.9) nor the 10-year OS rate (59.6% vs. 59.9%; *p* = 0.85) was meaningfully different [5]. Therefore, RT, whether delivered pre- or post-operatively or by short- or long-course duration, is unquestionably beneficial in terms of reducing locoregional recurrences in LARC patients. However, the locoregional recurrence (LRR) rate and the distant recurrence (DR) are still unacceptably high in patients with very high-risk features. The idea of administering upfront optimal systemic therapy, followed or preceded by either SC-RT or LC-CCRT prior to TME has been introduced. The new paradigm called total neoadjuvant therapy (TNT) has been tested in patients with high- to very-high-risk LARC. The updated results from the RAPIDO trial comparing SC-RT (5 × 5 Gy) followed by 6 cycles of CAPOX or 9 cycles of FOLFOX and TME with LC-CCRT (the standard of care, SOC) and TME demonstrated unexpected inferiority of TNT in terms of the LRR rate (10% in TNT vs. 6% in SOC, *p* = 0.027). Moreover, the 5-year OS rate was eventually insignificantly different (81.7% vs. 80.2%; *p* = 0.50) [6]. On the contrary, the updated results from PRODIGE-23 comparing 6 cycles of mFOLFIRINOX followed by LC-CRT, TME and another 6 cycles of mFOLFOX6 or 8 cycles of capecitabine with SOC revealed significant improvement of 7-year disease-free survival (DFS) from 62.5% to 67.6% (*p* = 0.048) and the 7-year OS rate from 76.1% to 81.9% (*p* = 0.033) [7]. The cross-comparison between these trials is not possible due to differences in the definition of very-high-risk lesions and their clinical end points. RT may potentiate long-term toxicities and affects a patient’s quality of life. The concept of omission of neoadjuvant RT has been proposed among patients with low- and intermediate-risk lesions. On the other hand, for LARC with low-and intermediate-risk features, some studies tested the concept of omitting neoadjuvant RT (neoadjuvant chemotherapy followed by TME) instead of the standard neoadjuvant with SC-RT with TME or LC-RT CCT with TME. For instance, the recent randomized phase 3 non-inferiority PROSPECT trial compared neoadjuvant mFOLFOX6 and selective LC-CRT (based on the trialists’ protocol) with the SOC. At a median follow-up of 58 months, the omission of pre-operative RT was noninferior to the SOC for DFS (5-year DFS of 80.8% vs. 78.6%, *p* = 0.005 for non-inferiority) and without compromises in OS and LRR [8]. To follow this new rectal cancer approach, it is necessary to define with clarity the indications of neoadjuvant therapy, the clinical definition of low-, intermediate-, and high-risk rectal cancers, the definition of good and poor responses to neoadjuvant therapy, the time to wait to assess neoadjuvant RT or chemoradiotherapy response, the space for the watch-and-wait strategy and the role of adjuvant chemotherapy in the different therapeutic options. None of these issues are completely clarified and are under debate. This review intends to reflect the actual state of the art of LARC treatment.

## 2. The Definition of Rectal Cancer

With due regard to the location of the rectum within the pelvic cavity and its close proximity with genitourinary organs, rectal cancer is subject to recur locally more often than cancer occurring in the more proximal part of the intestine. Furthermore, the addition of RT is not beneficial in lesions arising above the peritoneal reflection. The upper margin is defined by the NCCN as below a virtual line from the sacral promontory to the upper edge of the symphysis as determined by MRI, which approximately corresponds to 15 cm from the anal verge as measured by endoscopy [9]. An International Expert-based Delphi Consensus agreed that the “sigmoid take-off” is seen on computed tomography (CT) or MRI as an anatomic, image-based definition of the junction of the mesorectum and mesocolon. The sigmoid take-off is seen as the part of the colon that turns away from the sacrum and extends anteriorly, usually a few centimeters above the anterior peritoneal reflection [10]. Based on the moderate evidence from the ASTRO rectal cancer guideline, omission of neoadjuvant RT on the side of upfront surgery for patients with cT3a/bN0 when the lesion is located above 10 cm from the anal verge, predicted circumferential resection margin (CRM) ≥ 2 mm, and without extramural vascular invasion (EMVI) as determined by the high-precision pelvic MRI [11]. Practically, the definition of rectal cancer is based on its candidate for any neoadjuvant therapy, i.e., any tumor located within 12 cm from the anal verge.

## 3. Short-Course Radiotherapy (SC-RT) vs. Long-Course Concurrent Chemoradiotherapy (LC-CCRT)

Long-course concurrent chemoradiotherapy (LC-CRT) represents the conventionally fractionated RT, with doses of 180 to 200 cGy per fraction (Fr), administered in 25 to 28 daily fractions (five days per week) up to a total dose of 4500–5040 cGy, concomitant with capecitabine with a dose of 825 mg/m^2^ twice daily or 5-fluorouracil (5-FU) with a dose of 1200 mg/m^2^ daily. TME is usually scheduled 4–12 weeks after completion of the RT course, usually after 6th–7th week. Whether the administration of adjuvant chemotherapy is necessary remains controversial. Short-course radiotherapy (SC-RT) represents the hypofractionated RT, with doses of 5 Gy per fraction, administered in five daily fractions over the course of a week (5 × 5 Gy) without concomitant chemotherapy administration. Surgery is usually scheduled within the first 10 days after RT, most frequently in 5th to 7th day, or it can be delayed similar to LC-CRT. There are controversies and biases regarding which fractionation is more efficacious and less harmful. Theoretically, the large fraction sizes of the hypofractionation SC-RT injure both sphincter muscles and eventual post-operative anastomoses more than LC-CRT does. Notoriously, the rate of post-operative complications like leakage and fistula and long-term rate of permanent ostomies are imaginably higher. Most authorities believed that the LC-CRT is more suitable for high-risk lesions due to a better pCR rate. The delivery of short-course 5 × 5 Gy actually results in the biological effective dose (BED) of 66.7 Gy and the biological equivalent dose of 40 Gy [12]. The studies comparing neoadjuvant SC-RT and LC-CRT conducted by Bujko et al. [13] and Guckenberger et al. [14] revealed equal efficacies in terms of both locoregional control and survival. The long-term results of a phase III Polish II study also demonstrated no difference in OS and late complications between SC-RT and LC-CRT. Additionally, the investigators suggested that SC-RT was an alternative to chemoradiation due to lower acute toxicities, better cost-effectiveness and convenience [15]. The phase 3 Stockholm III study randomized 385 patients with resectable rectal cancer into three arms equally, SC-RT followed by immediate (within 1 week after RT) surgery vs. SC-RT with delayed (4–8 weeks after RT) surgery and LC-CRT with delayed surgery. The local recurrences occurred infrequently; on the contrary, DM was rather more common pattern of recurrences. The 5-year recurrence-free survival (RFS) rates were nearly identical (65%, 64%, 65%, respectively). Moreover, there was no significant difference in the 5-year OS rate (73%, 76% 78%, respectively). In terms of safety, even though SC-RT with delayed surgery was associated with more serious acute RT-induced toxicities (6.5% vs. 0.3%; *p* < 0.001) than SC-RT with immediate surgery was, 30-day post-operative complications (i.e., surgical-site infection, deep infection, anastomotic leak, post-operative bleeding, stoma-related complications, wound dehiscence, or other surgical complications) occurred less frequently (40.6% vs. 52.7%; *p* = 0.001) [16]. The meta-analysis conducted by Liscu et al. showed that SC-RT led to a more favorable short-term safety profile compared with LC-CRT, whereas the long-term toxicities were reported slightly more common with SC-RT. The most common acute adverse events (AEs) were mainly GI toxicities. Reports from earlier studies of SC-RT disclosed higher rate of permanent stroma was presumably owing to impaired healing (leakage, infection and fistula), stenosis or poor anorectal function. Among those who underwent sphincter-sparing surgery, SC-RT might accomplish fully recovered bowel and less bladder continence compared with LC-CRT. Nonetheless, pooled results from studies reporting the long-term quality of life revealed no difference between SC-RT and LC-CRT [17]. The differences in the pCR rate may be partly explained by the timing of surgery after completion of neoadjuvant RT. Noticeably, the fact that most patients treated with neoadjuvant SC-RT in earlier clinical trials usually underwent surgery immediately after RT would explain why those who had received pre-operative SC-RT achieved less tumor regression. The author recommends that whether SC-RT or LC-CRT is used, delayed surgery is preferable in the hope of a greater chance of tumor shrinkage.

## 4. The Optimal Time Interval between Neoadjuvant RT and Surgery

In the case of patients with high-risk LARC, neoadjuvant RT is indicated. Neoadjuvant (chemo)radiotherapy improves the pathological complete response (pCR) rate, the LRR-free rate and disease-free survival (DFS), even though it does not affect OS. Theoretically, a longer interval between RT and surgery would provide timing for maximal tumor regression, in expense of more fibrosis and tissue friability potentially resulting in worse TME quality and a higher complication rate. The pCR is usually an earlier surrogate of neoadjuvant treatment efficacy. The pCR rate is somewhat correlated with locoregional control; however, whether the pCR rate translates to superior survival outcomes remains a debatable issue. There have been signals from both retrospective and prospective randomized trials demonstrating that the longer interval from neoadjuvant (chemo)radiotherapy to surgery is associated with more substantial tumor regression. The phase 2 TIMING trial randomized 136 patients with clinical stage II–III rectal cancer who had received neoadjuvant LC-CRT into two groups, 66 patients in the short treatment delay (underwent surgery 6 weeks after CRT) group and another 70 patients in long treatment delay (if a patient achieved a clinical response 4 weeks after CRT, another 2 cycles of the mFOLFOX-6 was delivered and then underwent surgery 3–5 weeks later) group. The average time from CRT-to-surgery was 6 and 11 weeks, respectively. The pCR rate was higher in long treatment delay group compared with the short delay group (25% vs. 18%); however, the difference did not reach statistical significance. The pCR rate for the patients in the long treatment delay group who had received 2 cycles of mFOLFOX-6 was also 25%. The proportion of patients who had a sphincter-preserving low anterior resection, and an R0 resection, as well as average number of lymph nodes obtained in surgical specimens, were similar in both groups. The average pelvic fibrosis score was higher in the long treatment delay group, but surgical difficulty was identical in both groups. Equivalent rates of severe AEs during neoadjuvant therapy were also noted [18]. Due to its small sample size, the statistical results should be considered judiciously. The phase 3 GRECCAR6 revealed the contradictory results. The investigators recruited 265 patients who had received LC-CRT and randomized the participants into the 7-week delay arm and the 11-week delay arm. The pCR rate was not different (15.0% vs. 17.4%; *p* = 0.5983). Unexpectedly, compared with those in the shorter delay arm, those in the longer delay arm experienced more from medical complications (19.2% vs. 32.8%, *p* = 0.0137) and had a worse quality of mesorectal resection (complete mesorectum rate of 90% vs. 78.7%; *p* = 0.0156) [19]. The phase 3 Stockholm III trial is one of the landmark clinical trials that compared the efficacies between both different RT fractionation regimens and the effect of time interval between neoadjuvant RT and surgery on the pCR rate. The combined analysis of both three-armed (SC-RT with immediate surgery, 129 patients vs. SC-RT with delayed surgery, 128 patients vs. LC-CRT with delayed surgery, 128 patients) randomization and two-armed (SC-RT with immediate surgery, 288 patients vs. SC-RT with delayed surgery, 227 patients) randomization reported substantially more tumor regression after SC-RT with delayed surgery. The pCR rate was observed in 0.3%, 10.4% and 2.2% of patients in SC-RT with immediate surgery, SC-RT with delayed surgery and LC-CRT with delayed surgery, respectively. Moreover, those who fulfilled pCR and Dworak grade 4 tumor regression tended to have superior survival and longer time to recurrence (TTR) (pCR vs. no-pCR HR for OS, 0.51; 95% CI, 0.26–0.99; *p* = 0.046 and HR for TTR, 0.27; 95% CI, 0.09–0.86; *p* = 0.027) [20]. The updated survival data with median follow-up of 5.7 years revealed comparable OS. Median OS in the three-armed randomization group was 8.1 (range 6.9–11.2), 10.3 (range 8.2–12.8), and 10.5 (range 7.0–11.3) years after SC-RT with immediate surgery, SC-RT with delayed surgery and LC-CRT with delayed surgery, respectively. Median OS in the two-armed randomization group was 8.1 (range 7.2–10.0) and 10.2 (range 8.5–11.7) years after SC-RT with immediate surgery and SC-RT with delayed surgery, respectively. In addition, there was no statistically significant difference in health-related quality of life (HRQoL) [21]. Since the Stockholm III study was designed in non-inferiority and unblinded fashion, the comparisons must be interpreted cautiously. Akgun et al. also randomized 327 patients who had received LC-CRT into the short treatment delay (surgery performed within 8 weeks after CRT) group and long treatment delay (surgery performed 8 weeks or more after CRT) groups. The long interval group achieved a higher pCR rate (10% vs. 18.6%; *p* = 0.027). Fascinatingly, the highest pCR rate (29%) was detected between 10 and 11 weeks. Statistically significant tumor regression in the long treatment delay group and better composite post-neoadjuvant pathological (yp) stage and ypT category were also observed. The concerning surgical quality (rates of tumor-positive margins, TME quality, anastomotic leakage and intraoperative perforation) between both groups were also similar [22]. Amin et al. conducted a retrospective analysis using data from the US National Cancer Database (NCDB) to determine the optimal time interval between neoadjuvant RT and surgery associated with an improved pCR rate. They reported that comparing with less than 4 weeks after RT, the longer interval was co-related with the better pCR rate. The proportion of patients who achieved pCR increased from 9% for those who underwent surgery within 4 weeks after RT to 14%, 18%, 16% and 15% for those who underwent surgery between 5 and 8 weeks, 9 and 12 weeks, 13 and 16 weeks and 17 and 24 weeks after RT, respectively. Patients who achieved pCR had a longer OS compared with those who did not. Interestingly, those who achieved pCR had comparable OS regardless of when the surgery performed between ≤4 and 24 weeks after finishing RT. However, when compared to the overall population of subjects, those who had surgery ≤ 12 weeks after RT had a higher OS compared with those who had surgery > 12 weeks after RT [23]. Based on this study, the optimal time interval to undergo surgery after neoadjuvant (chemo)radiotherapy should be approximately 9–12 weeks after RT.

The recent randomized phase 2 OPRA study compared induction chemotherapy (IC) (either 8 cycles of FOLFOX or 6 cycles of CAPOX) and then LC-CRT (induction TNT group) with consolidation chemotherapy (CC) after LC-CRT (consolidation TNT group). According to the study protocol, standard watch-and-wait (WW) strategy was offered, if a patient had achieved pCR after tumor restaging obtained within 8 (±4 weeks) weeks after completion of TNT. Whether the fact that the median time from completion of CRT to assessment of treatment response was longer in the consolidation TNT group (28.5 weeks) compared with the induction TNT group (8 weeks) would explain the higher proportion of patients who preserved the rectum at 3 years in the consolidation TNT group compared with the induction TNT group (53% vs. 41%; *p* = 0.01), corresponding to the lower rate of tumor regrowth in the consolidation TNT group compared with the induction TNT group (27% vs. 40%) was an arguable issue. The investigators also found that the 3-year probability of organ preservation was better for patients with clinical complete remission (CCR) compared with those with non-CCR (77% vs. 40%, *p* = 0.001). Clinical tumor response grade was associated with DFS, local recurrence-free survival, distant metastasis-free survival, and OS [24]. The longer time since completion of LC-CRT to tumor assessment would attribute to more accurate assessment of tumor shrinkage.

In conclusion, the longer interval between RT and surgery contributes to higher degree of tumor regression. The optimal time to assess tumor response is between 8 and 12 weeks after the completion of RT. Whether systemic therapy should be added while waiting for the operation (consolidation chemotherapy) to optimize the survival outcomes remains disputable.

## 5. Total Neoadjuvant Therapy (TNT): Data from the Clinical Trials

Patients with high- to very-high-risk LARC have more chances in developing both LRR and DM compared with those with less extensive tumor invasion. Theoretically, the earlier exposure to the most efficacious systemic chemotherapy either before (induction) or after (consolidation) neoadjuvant (chemo)RT would eradicate the micro-metastasis promptly. Over and above, systemic chemotherapy would potentiate the RT effect in tumor downsizing. Furthermore, the compliance would be better, if systemic chemotherapy is administered during pre-operative period compared with post-operative one. There are many TNT protocols that have been developed and proposed for high- to very-high-risk LARC. Different protocols selected patients with different characteristics of local invasiveness and used different sequences between systemic therapy and (chemo)RT. Moreover, different systemic therapy and different RT regimens might affect and confound the outcomes. Which end point should be the ultimate one is also a disputable issue. The LRR-free rate would be the logical end point for those with very-high-risk lesions who distant recurrences would be naturally unavoidable. The salvage therapy for such patients with LRR like pelvic exenteration may be unamenable or unachievable without burdensome morbidities. On the contrary, for those with moderate-to-high-risk lesions, the LRR-free rate, DFS and OS are equally noteworthy. The pCR rate is the quite popular surrogate of survival outcomes of neoadjuvant therapy because it requires a much shorter time of follow-up. A literature-based meta-analysis of randomized clinical trials conducted by Petrelli et al. demonstrated that both the pCR rate and 3-year DFS were not reliable surrogates for treatment effects on the 5-year OS rate (R = 0.2; 95% CI, −0.29–0.78; *p* = 0.5 and R = 0.64; 95% CI, 0.29–1; *p* = 0.06, respectively) [25]. The neoadjuvant rectal (NAR) score has been developed and proposed. It uses clinical T stage, clinical and pathological N stages as the necessitated factors to be calculated. The calculated scores stratify the patients into 3 risk groups, low, intermediate and high risk of mortality [26].

Table 1 showed the summarized updated data from the recent TNT trials. Notably, the effect of TNT on DFS and OS seems to be faded gradually upon longer time of follow-ups.

The Polish II trial is a phase 3 trial recruiting 515 patients with fixed clinical T3 (cT3) or cT4 rectal cancer. The study randomized between SC-RT (5 × 5 Gy then 3 cycles of FOLFOX4, the experimental TNT group) and LC-CRT (50.4 Gy/28 Fr concomitant with bolus 5-FU, leucovorin and oxaliplatin, the control group). Surgery was performed about 12 weeks after initiation of RT and about 6 weeks after neoadjuvant therapy. The adjuvant chemotherapy (AC) with FOLFOX regimen was optional upon a clinician’s discretion. The study did not reach the primary end point of the R0 resection rate. The Ro resection rates in the experimental TNT group and the control LC-CRT-only group were 77% and 71%, respectively (*p* = 0.081). The pCR rate in the TNT group was only 19%, not different from the control group that achieved only 11.5% (*p* = 0.19). The 3-year OS (73% vs. 64.5%; *p* = 0.055), DFS (53% vs. 52%; *p* = 0.74) and local failure rate (22% vs. 21%; *p* = 0.82) were also not significantly different. Both post-operative (29% vs. 25%, *p* = 0.18) and late complications (20% vs. 22%, *p* = 0.54) rates were also comparable [27]. The updated 8-year OS (49% vs. 49%) and DFS (43% vs. 41%; *p* = 0.65) were almost identical. The local failure rate (35% vs. 32%; *p* = 0.60) and distant metastatic rate (36% vs. 34%; *p* = 0.54) were almost equivalent. Astonishingly, the rate of serious late AEs was also similar (11% vs. 9%; *p* = 0.66) [15]. The Polish II study confirmed the non-inferiority of SC-RT in terms of efficacies in downsizing tumor, survival outcomes and safety; however, it did not prove the necessity of systemic chemotherapy administered during the neoadjuvant period. The CAO/ARO/AIO-12 trial is a phase 2 trial enrolling 306 patients with clinical stage 2 and 3 rectal cancer. The study allocated the participants into two different TNT regimens, the induction chemotherapy (IC) arm (3 cycles of FOLFOX then LC-CRT, 50.4 Gy delivered in concomitant with 5-FU/oxaliplatin) and the consolidation (CC) arm (LC-CRT then 3 cycles of FOLFOX). Its primary end point was the pCR rate. Not only the longer interval between completion of CRT and surgery in the consolidation TNT arm (median of 90 vs. 45 days) did not worsen surgical morbidity, but also the pCR rate was improved (from 17% in the induction TNT arm to 25% in the consolidation TNT arm). Therefore, the consolidation TNT was the better regimen. Again, the CAO/ARO/AIO-12 did not prove the necessity of systemic chemotherapy during the pre-operative period. Extending the period from completion of RT to surgery would explain the better pCR rate [28]. The OPRA trial is a phase 2 trial recruiting 324 patients with low-lying (within 5 cm above the anal verge) clinical stage II or III rectal adenocarcinoma. The study compared two different TNT regimens, the induction TNT (8 cycles of mFOLFOX6 or 5 cycles of CAPOX prior to LC-CRT, 50–56 Gy concomitant with either continuous infusion 5-FU or capecitabine) and the consolidation TNT (LC-CRT prior to the same chemotherapy regimen). The patients were allowed to participate in a standardized watch-and-wait (WW) protocol, if they accomplished cCR or near-cCR as determined by the tumor restaging work-ups obtained within 8 (±4) weeks after TNT. The study aimed to prove the superiority of TNT in terms of DFS compared with the historical benchmark of 75% 3-year DFS. Even though the higher rate of tumor regrowth in the induction TNT group (40%) compared with the consolidation TNT group (27%), both 3-year DFS (76% vs. 76% for induction vs. consolidation TNT) and 3-year TME-free survival (41% vs. 53%) were similar and not significantly different from the historical benchmark. In addition, no difference was detected between groups in terms of LRR-free survival, DM-free survival, and OS. Only clinical nodal metastasis (cN+) on baseline MRI was the significant predictor of adverse DFS. No matter the systemic chemotherapy was administered before or after LC-RT, the proportion of patients received protocol-defined number of systemic chemotherapy cycles was similar. Notably, the fact that almost 10% of patients undergoing TME had a pCR would reflect the inaccuracy of the post-neoadjuvant tumor response assessment. The investigators concluded that organ preservation was attainable in half of the participants, without any detrimental effect in survival outcomes [29]. The updated data revealed insignificant 5-year DFS between both groups (71% vs. 69%; *p* = 0.68); however, 5-year TME-free survival was better in the consolidation TNT arm (39% vs. 54%, *p* = 0.012). Provocatively, either patients who underwent TME after restaging or after regrowth had identical 5-year DFS rates (64% vs. 64%; *p* = 0.94). The tumor regrowth almost appeared within the first 3 years after treatment [24]. Even though the study did not reach its primary end point, the organ preservation rate in the OPRA trial was notably higher than the pCR rates reported in previous clinical trials. Whether the long interval from the end of TNT to the tumor response assessment and allowance of WW strategy for patients with near-cCR response could explain such findings remains inconclusive.

The RAPIDO and the UNICANCER PRODIGE 23 trials are the landmark phase 3 studies specifically designed to prove the clinical benefits of TNT over the conventional neoadjuvant (chemo)RT among patients with high- to very-high-risk LARC based on the state-of-the-art imaging study.

The RAPIDO trial recruited patients with one of the following criteria, clinical tumor [cT] stage cT4a or cT4b, extramural vascular invasion (EMVI+), clinical nodal [cN] stage cN2, involved mesorectal fascia (MRF+), or enlarged lateral lymph nodes as determined by pelvic MRI. There were 920 patients enrolled and 1:1 randomly assigned to either the experimental TNT group (SC-RT followed by 6 cycles of CAPOX or 9 cycles of FOLFOX4 followed by TME with a chemotherapy-free interval between days 3 and 14 with or without adjuvant CAPOX or FOLFOX4 per physician’s choice or hospital’s policy) or the standard of care group (SOC) (LC-CRT, 50.4 Gy/28 Fr or 50.0 Gy/25 Fr) with concomitant capecitabine followed by TME with or without AC (8 cycles of CAPOX or 12 cycles of FOLFOX4 per physician’s choice or hospital’s policy). Approximately one-third of participants had cT4, two-thirds had cN2, two-thirds had MRF+ and one-third of participants had tumor 10 cm or more above the anal verge were noted. In the TNT group, median time between completion of RT to commencement of CAPOX was 14 days. The overall treatment time prior to surgery was approximately 40 weeks in the TNT group compared with approximately 25 weeks in the SOC group. The study reached its primary end point of improvement in 3-year disease-related treatment failure (DrTF, defined as the first occurrence of locoregional failure, distant metastasis, new primary colorectal tumor, or treatment-related death). The DrTF occurred significantly more frequently in the SOC group compared with the TNT group (30.4% vs. 23.7%; HR 0.75; 95% CI, 0.60–0.95; *p* = 0.019). More serious adverse events during neoadjuvant therapy were reported in the TNT group (48%) compared with the SOC group (25%). Colitis was the most common serious events in both groups. TNT doubled the pCR rate (28% vs. 14%; *p* < 0.0001); however, both the R0 resection rate and the rate of surgery with a close or involved circumferential radial margin (CRM+) were similar. At 3 years, DM was the most frequent pattern of failure. Fewer patients in the TNT group experienced DM (20% vs. 26.8%; HR 0.69; 95% CI, 0.54–0.90; *p* = 0.048). The investigators performed the sensitivity analysis to determine the effect of adjuvant post-operative chemotherapy on 3-year DrTF and revealed no association [30]. Paradoxically, the updated data after median 5.6 years of follow-up disclosed more common loco-regional recurrence (LRR) in the TNT group compared with the SOC group (10% vs. 6%; *p* = 0.027), even though the good responders as determined by the post-treatment restaging were declared more often among those received TNT compared with the SOC (80.1% vs. 70.1%; *p* < 0.0001). Both groups developed LRR at nearly the same time within the first 2 years after treatment. Median duration from surgery to the detection of an LRR were 1.8 years in the TNT group and 1.2 years in the SOC group (*p* = 0.31), respectively. Interestingly, when an LRR was detected, half of them had prior or synchronous DM, nearly identically in both groups (50% in the TNT group vs. 54% in the SOC group; *p* = 0.84). Salvage resection was performed in 50% patients in the TNT group and 42% patients in the SOC group who had had local recurrence, most (82%) with the curative intent. The exploratory post hoc analysis reported further astonishing findings. Regarding the RT technique, those who had received 3D-CRT in the TNT group developed LRR more often than those who had received the same technique in the SOC group (11.6% vs. 6.0%; *p* = 0.016), while those who had received intensity-modulated radiation therapy (IMRT) or volumetric-modulated arc therapy (VMAT), no difference in LRR between those in TNT group and those in the SOC group (6.3% vs. 6.2%, *p* = 0.96) was reported. Actually, patients in the TNT group had received more often 3D-CRT, whereas the SOC group had received IMRT/VMAT more. Enthusiastically, TNT led to worse TME quality as defined as an intraoperative breach of the mesorectum (11% in the TNT group vs. 6% in the SOC group; *p* = 0.022). Moreover, among those with breached mesorectum, LRR was more often occurred in the TNT group (21% vs. 4%; *p* = 0.053). Unexpectedly, in addition to predictable adverse prognostic features like enlarged lateral lymph nodes, CRM+, tumor deposits, and node positivity at pathological specimens, the TNT paradigm was also an independent predictor of LRR. At 5 years, the superiority of TNT in terms of the cumulative probability of DrTF remained unchanged (27.8% in the TNT group vs. 34.0% in the SOC group; HR 0.79; 95% CI, 0.63–1.00; *p* = 0.0480), as well as the cumulative probability of DM in the TNT group 23.0% vs. 30.4% in the SOC group (HR 0.73; *p* = 0.011). Nonetheless, the 5-year OS rate was not significantly improved with TNT (81.7% in the TNT group vs. 80.2% in the SOC group (*p* = 0.50) [6]. Whether the ATRESS (neoadjuvant Therapy-RElated Shortening of Survival) phenomenon could explain the fact that improvement in DrTF did not transfer to the OS benefit remains speculative. Since the AC was not mandatory, only 59% of patients, equally in both groups, received the AC. The early exposure to the standard combination chemotherapy during the pre-operative period would result in developing the chemo-resistant clones. Therefore, no further chemotherapy regimen was effective, if the DM emerged or else when the DM appeared, it would behave differently and more aggressively. Disappointingly, the LRR eventually occurred more often in the TNT group, even though there were more good responders in this group would be partly explained by the fact that the surgeons were persuaded by the results from the post-treatment assessment. Therefore, there were tendencies to perform the sub-optimal operations like sphincter-sparing surgery in the TNT group. The investigators suggested that the extent of surgery and the surgical technique should be determined based on the pre-treatment evaluation, instead.

The UNICANCER-PRODIGE 23 trial recruited 461 patients with cT3 (at risk of local recurrence as judged by the multidisciplinary tumor board) or cT4 rectal cancer and 1:1 randomly assigned to either TNT consisting of FOLFIRINOX (irinotecan 180 mg/m^2^, oxaliplatin 85 mg/m^2^, leucovorin 400 mg/m^2^, and fluorouracil 2400 mg/m^2^ 46-h continuous infusion) for 6 cycles followed by LC-CRT (50 Gy/25 Fr concomitant with capecitabine) within 1–3 weeks after the last chemotherapy cycle or the SOC (LC-CRT). Standard surgery including TME was mandatory (watch-and-wait policy was not allowed) and performed within 6–8 weeks after CRT. AC (6 cycles of mFOLFOX6 or 4 cycles of capecitabine in the TNT group and 12 cycles of mFOLFOX6 or 8 cycles of capecitabine in the SOC group) was also strongly recommended and started within 5–12 weeks after surgery, regardless of pathological response stage. There were 13% of patients with tumor located at 10 cm or more above the anal verge (AV). Eighteen percent of patients in the TNT group and 16% in the SOC group had cT4 (most had cT4b). Approximately one-fourth of both groups had cN2 and one-fourth of them had MRF+ (less than 1 mm predicted radial mesorectal margin). After the 46.5 months of median follow-up, the study reached its primary end point of improvement in 3-year DFS rate (76% in the TNT group vs. 69% in the SOC group (HR 0.69; 95% CI, 0.49–0.97; *p*=0.034); however, the 3-year OS was comparable (91% vs. 88%). TNT doubled the pCR rate (28% vs. 12%, *p* < 0.0001) without significant difference in mesorectal excision quality. The R0 resection rate was identical (95% vs. 94%). The definitive colostomy rate was also similar (14% vs. 15%). Approximately three-thirds of the participants received AC. More patients in the SOC group received at least 80% of the planned doses (22% in the TNT group vs. 51% in the SOC group; *p* < 0.0001); however, they had significantly more treatment delays and received less cumulative oxaliplatin dose overall. No significant difference in rates of serious AEs between both groups was detected (27% vs. 22%; *p* = 0.167) during the whole treatment period. LRR occurred infrequently in both groups (4% vs. 6%). DM was the most common pattern of failure. Again, TNT prevented more DM than the SOC (17% vs. 25%). Only TNT and lower clinical TNM stage were the significant predictor of longer DFS [31]. The updated analysis after a median follow-up of 82.2 months, the improvement in DFS rate in the TNT arm was retained (5-year DFS 67.6 vs. 62.5%; *p* = 0.048). More patients in the TNT group survived. The 5-year OS rate was eventually significantly diverged (81.9% vs. 76.1%; *p* = 0.033). TNT also significantly boosted the metastatic-free survival (MFS) as well (73.6% vs. 65.4%; *p* = 0.011) [7]. The comparison with the RAPIDO trial is not possible since the participants in the PRODIGE 23 had less very-high-risk features (fewer patients with cT4, cN2 and MRF+). Furthermore, the number of patients with enlarged lateral lymph nodes was not reported. The study was not designed to prove the contribution of the intensified triplet chemotherapy FOLFIRINOX on survival benefits.

The most recent STELLAR trial from mainland China recruited 599 patients with distal or middle-third, cT3 or cT4 and/or cN+ (as determined by MRI) rectal cancer and 1:1 randomly assigned to either TNT (consisting of SC-RT (5 × 5 Gy) followed by 4 cycles of CAPOX) or the SOC (consisting of LC-CRT (50 Gy/25 Fr concomitant with capecitabine). TME was performed within 6–8 weeks after neoadjuvant therapy, with 2 additional cycles of CAPOX in the TNT group and 6 cycles of CAPOX in the SOC group. Approximately one-sixth of the participants had cT4, one-third of them had cN2, half of them had MRF involvement and most of them had clinical stage 3 (85.8% and 83.5% in the TNT group and the SOC group, respectively). After a median follow-up of 35.0 months, the study proved its primary end point of non-inferiority in 3-year DFS (64.5% in the TNT group vs. 62.3% in the SOC group (HR 0.883; 1-sided 95% CI, NA to 1.11; *p* < 0.001 for noninferiority). The Ro resection rates were comparable (91.5% vs. 87.8%; *p* = 0.189); however, TNT nearly doubled the pCR rate (21.8% vs. 12.3%, *p* = 0.002). LRR was uncommon (6.6% vs. 7.7%); on the contrary DM was the much more common pattern of recurrences (21.5% vs. 22.6%). Even though there was no significant difference in both MFS and LRR, the TNT group had a strong inclination to improve the 3-year OS (86.5% vs. 75.1%; *p* = 0.033). Only the low-lying tumor (within 5 cm from anal verge) had a tendency towards improvement in DFS and OS with TNT, while other baseline characteristics were not the prognostic factors. Nonetheless, the rate of serious acute AEs during pre-operative treatment was significant higher in the TNT group compared with the SOC group (26.5% vs. 12.6%; *p* < 0.001), mostly from hematologic toxicities during the period of IC. Among those (76% of all participants) who went on to surgery, one-fourth of them in each group did not receive AC. Of those who received AC, more patients in the TNT group completed planned cycles (60.0% vs. 48.3%; *p* = 0.009) [32]. Due to its non-inferiority design, it is not logical to deduce the clinical benefits of TNT. Moreover, the survival data are still immature. Comparing with the PRODIGE 23 trial, the STELLAR trial recruited more patients with very-high-risk features (cN2 and MRF+). This fact would explain why the 3-year DFS of the STELLAR trial was inferior to the PRODIGE 23. Notably, the STELLAR trial excluded those with lesions at upper rectum. Its TNT protocol was quite similar to the TNT protocol of the RAPIDO trial except fewer number cycles of consolidation chemotherapy (4 vs. 6 cycles). Again, the RAPIDO trial recruited more patients with very-high-risk features than the STELLAR trial. The pCR rate in the RAPIDO trial was slightly higher compared with the STELLAR trial (28% vs. 21.8%). Whether more cycles of CC contributed to a better pCR rate was another debatable issue. The number of patients with enlarged lateral lymph nodes was not stated in the STELLAR trial similar to the PRODIGE 23 trial.

In conclusion, TNT does improve the pCR, but whether the improvement was attributed by the CC or longer time from RT to surgery is an issue to be further explored. Since the DM is consistently more common than LRR, a combination chemotherapy regimen should be incorporated. Whether the doublet FOLFOX/CAPOX or more intensified triplet FOLFIRINOX is the best companion after RT is a challenging question. Whichever chemotherapy regimen is used, it should be prescribed after RT (consolidation TNT), while a patient is waiting for time to maximal tumor regression. Because the OS benefit might be confounded by the number of chemotherapy cycles received during both pre-operative and post-operative periods, until there is a randomized clinical trial specifically designed to explore the benefits of AC after TNT, the author suggests that among LARC patients with very-high-risk features need AC, regardless of their degrees of tumor regression. To avoid suboptimal surgery, a surgeon should determine the extent and technique of surgery based on the findings from the pre-treatment evaluation. Watch-and-wait policy is not recommended among patients with very-high-risk features even though they can achieve cCR. Whether the pre-operative SC (5 × 5 Gy) or LC-CRT regimen should be applied is judged by an institute’s experiences and a patient’s preference. Whatever RT regimen used, it should be delivered by the highly conformal technique (IMRT or VMAT). Given that the substantial toxicities with such intensified TNT protocols are unavoidable, pre-treatment staging evaluation with MRI using the state-of-the-art protocol with or without adjunctive endo-rectal ultrasonography is compulsory in order to precisely select the most proper candidates for TNT.

**Table 1:** Showed the summarized study schema of the published TNT trials and their outcomes.

Study (Year) (Phase; Primary End Point)	Inclusion Criteria	Schema	Adjuvant Therapy	N	Pcr Rate	R0 Resection Rate	DFS/ LRR	OS	Remarks
POLISH II (2019) [15] (Phase 3; R0 resection rate)	cT3/cT4	**EXP:** RT 5 × 5 Gy → 3 × FOLFOX → Surgery (6 weeks later)	Ox-based (Optional)	261	16%	77%	43%/(LFR35%) (8-year)	49% (8-year)	Less acute toxicities in the EXP group. Similar late complications.
		**CTL:** CRT 50.4 Gy with FU/OX → Surgery (6 weeks later)		254	11.5%	71%	41%/(LFR32%) (8-year)	49% (8-year)	
CAO/ARO/AIO-12 (2019) [28] (Phase 2; pCR rate)	Clinical stage II-III	**IND:** 3 × FOLFOX → CRT 50.4 Gy with FU/OX		156	17%		73%/6% (3-year)		Worse compliance with consolidation chemotherapy. No difference in late toxicities.
		**CSL:** CRT 50.4 Gy with FU/OX → 3 × FOLFOX		150	25% *		73%/5% (3-year)		
OPRA (2020) [24] (Phase 2, DFS)	Clinical stage II-III (Watch and wait (WW) was offered, if a patient achieved clinical CR or near CR after TNT.)	**IND:** 8 × FOLFOX or 6 × CAPOX → CRT 50–56 Gy with FU C.I. or Capecitabine		158			71% (5-year)	(TME-free survival: 39%)	Similar (64%) DFS rate in both groups of patients who underwent TME after restaging and patients who underwent TME after tumor regrowth. Long-term organ preservation was accomplished in half of the patients.
		**CSL:** CRT 50–56 Gy with FU C.I. or Capecitabine → 8 × FOLFOX or 6 x CAPOX		166			69% (5-year)	(TME-free survival: 54%)	
RAPIDO (2020) [6,30] (Primary end point: disease-related treatment failure)	cT4a or cT4b, extramural vascular invasion, cN2, involved mesorectal fascia, or enlarged lateral lymph nodes	**EXP (TNT):** 5 × 5 Gy → 6 × CAPOX or 9 × FOLFOX → Surgery (within 3–14 days after last course of chemotherapy)		462	28.4% *		(5-year disease-related treatment failure: 27.8%) (LRR 10%, LRF 12%)	81.7% (5-year)	More grade ¾ diarrhea occurred during pre-operative treatment in the EXP group. OS after LRF was comparable.
		**CTL:** CRT 50.4 Gy/25 Fr or 50 Gy/20 Fr concomitant with capecitabine → Surgery (after 8 ± 2 weeks after RT)	8 × CAPOX or 12 × FOLFOX (optional)	450	14.3%		(5-year disease-related treatment failure: 34%) * (LRR 6%, LRF 8%)	80.2% (5-year)	
PRODIGE-23 (2020) [7,31] (Primary end point: DFS)	cT3 or cT4	**EXP (TNT):** 6 × FOLFIRINOX → CRT 50 Gy concomitant with capecitabine → Surgery	3 months of mFOLFOX6 or CAPOX	231	27.8% *		67.6% */ 5.3% (7-year)	81.9% * (7-year)	Insignificant differences in serious adverse events between both arms during the whole treatment period. Neoadjuvant chemotherapy could relieve tumor-related symptoms.
		**CTL:** CRT 50 Gy concomitant with capecitabine → Surgery	6 months of mFOLFOX6 or CAPOX	230	12.1%		62.5%/ 8.1% (7-year)	76.1% (7-year)	
STELLAR (2022) [32] (Primary end point: DFS)	cT3 or cT4 and/or cN+	**EXP (TNT):** 5 × 5 Gy → 4 × CAPOX → Surgery (within 6–8 weeks after finishing pre-operative treatment)	2 × CAPOX	302	21.8% *		64.5% (3-year)	86.5% * (3-year)	More serious acute toxicities during pre-operative therapy in the TNT group.
		**CTL:** CRT 50Gy/25Fr concomitant with capecitabine → Surgery (within 6–8 weeks after finishing pre-operative treatment)	6 × CAPOX	297	12.3%		62.3% (3-year)	75.1% (3-year)	

* statistically better; EXP, experimental; CTL, control; IND, induction; CSL, consolidation; TNT, total mesorectal excision; DFS, disease-free survival; LRF, locoregional-failure rate; LRR, locoregional recurrence rate.

## 6. Which Patients Deserve Most from the TNT?

Besides some degrees of unavoidable GI toxicities from RT and direct injuries of RT to adjacent tissues potentially affecting the surgical quality, TNT incorporates a potent combination chemotherapy subject to both GI and hematologic adverse events that would lead to detrimental effects in delaying subsequent therapies and unexpected fatal events. Consequently, TNT should be recommended for patients with LARC harboring high- to very-high-risk features. Although, any TNT protocols did not compromise subsequent treatments including surgical intervention based the results from the phase 2 and phase 3 trials, to avoid overtreatment in patients with low-risk cT3 or undertreatment in patients with undetected perirectal or lateral pelvic lymph nodes, the most precise pre-treatment clinical staging evaluation is necessary. The high-resolution pelvic MRI is indispensable in accomplishing two essential goals. Firstly, to identify tumors at high risk of LRR that could benefit from neoadjuvant (chemo)radiotherapy. Among rectal patients with MRF+, how to obtain tumor-free circumferential resection margin (CRM) is the primary surgical trajectory [33]. Secondly, to detect lesions with higher distant metastatic potential. Earlier exposure to an optimal systemic therapy pre-operatively would result in preventing DM. The MRF has a strong potential to develop local recurrence, while involvement of the anterior peritoneal reflection is closely associated with the chance of peritoneal dissemination. Distinctively, the risk of MRF invasion by lower rectal tumors is extremely higher, with surgical margins positive in up to 30% of cases [34]. The MERCURY (Magnetic Resonance Imaging and Rectal Cancer European Equivalence) group is a pivotal trial establishing MRI-based prognostic groups that significantly impact LRR rates. Its 5-year follow-up revealed that MRI-involved MRF (MRF+) was the only pre-operative staging parameter that independently associated with adverse OS (62.2% in MRF- vs. 42.2% in MRF+, HR 1.97; 95% CI, 1.27 to 3.04; *p* < 0.01), DFS (67.2% vs. 47.3%, HR 1.65, 95% CI, 1.01 to 2.69; *p* < 0.05), and LRR (HR 3.50, 95% CI, 1.53 to 8.00; *p* < 0.05) [35]. The OCUM study used the high-resolution MRI in determining clinical T- and N categories, clinical stage and minimal distance between the tumor and mesorectal fascia (mrMRF) among 609 rectal cancer patients to compare with the histopathological categories as reported from the resected specimens. The mrMRF and circumferential resection margin (CRM) were regarded as tumor free at a minimal distance of less than 1 mm. The T category was correct in 63.5% of patients; cT was over-staged in 22.9% and under-staged in 13.5%. MRI accuracy for pathological lymph node involvement was 56.5%. Astonishingly, the substantial number of patients with clinical stage II (22.2%) and stage III (28.1%) were diagnosed by histopathology as stage I. The accuracy for tumor-free CRM was 86.5% and the NPV was 98.1%. Only 1.7% of patients had false negative mrMRF [36].

Most of the world authorities have agreed on the definition criteria of high-risk features that include (1) the MRF invasion (MRF+), (2) presence of extramural vascular invasion (EMVI+), (3) tumor deposits (TDs), (4) lymph node infiltration, (5) intrinsic tumor factors such as proximity or infiltration of the anal canal and the sphincteric apparatus, and (6) presence of mucin [37]. Based on the most recent Rectal cancer lexicon 2023 [9], the Society of Abdominal Radiology Colorectal and Anal Cancer Disease-Focused Panel has revised and updated the consensus statement. These are the summarized some of the notable definitions of the high-risk features.

- cT3-4 (for MRI): The cT3 tumor extends beyond the muscularis propria to involve mesorectal fat. It is further grouped into two prognostic categories, the T3 a/b (“good prognosis”) with up to 5 mm extramural invasion; and T3 c/d (“higher risk of local recurrence”) with more than 5 mm invasion beyond the muscularis propria. Any T3 substage with MRF+ is also associated with a higher risk of local recurrence. The cT4a denotes the peritoneal invasion. The cT4b includes the involvement of adjacent pelvis organs, including the uterus, ovaries, vagina, prostate, seminal vesicles, bladder, ureters, ureter, bone, and skeletal/striated muscular structures. Additionally, recent expert consensus also recommended assigning a T4b category, specifically if the tumor involves the following structures: extra-mesorectal vessels, sciatic or sacral nerves, sacrospinous/sacrotuberous ligaments, soft tissue beyond the mesorectum, such as fat of the obturator, iliac, or ischiorectal space and external anal sphincter. Of noted, involvement of the MRF, anterior peritoneal reflection, internal anal sphincter, or inter-sphincteric space does not constitute T4b.

Due to low CRM+ rate and favorable prognosis as rectal cancer patients with cT2 lesion, the ASTRO Clinical Practice Guideline suggests that neoadjuvant radiotherapy may be omitted for those with cT3a/b lesions on the condition that they are located > 10 cm from the anal verge with predicted CRM ≥ 2 mm and absence of EMVI as determined by MRI [11].

- Suspicious pathological lymph node involvement: The locoregional lymph nodes include mesorectal, superior rectal, and inferior mesenteric nodes (superior to the take-off of the left colic artery from the inferior mesenteric artery). If the tumor extends below the dentate line, inguinal lymph nodes are also determined as locoregional lymph nodes.
- Suspicious lateral pelvic side lymph nodes: Based on the historical anatomic studies that demonstrated lymphatic drainage from the lower rectum (below the peritoneal reflection) also passes to the internal iliac and obturator spaces. Among those with cT1/2 tumors or with higher lesions, the propensity of lateral pelvic lymph node involvement is low. Specifically for the internal iliac and obturator lymph nodes, a size of >7 mm in the short axis is considered suspiciously pathologic. Also noted, this criteria is applicable in the setting of cT3/4 tumors located < 8 cm from the AV. The reported prevalence was approximately 10–25% of patients with LARC, almost always found in those with low-rectal cancer, in particular [38].
- Suspicious non-locoregional/distant lymph nodes: For external iliac, common iliac, paraaortic, and inguinal nodes (if the rectal cancer is above the dentate line) of >10 mm in the short axis are considered suspicious non-locoregional/distant lymph nodes (metastatic disease or M1) in the setting of rectal cancer.
- Clinical nodal classification: If N classification is established based on findings from pelvic MRI, the consensus recommends using “cN+” for suspicious locoregional lymph nodes and/or tumor deposits and “cN−” for the absence of any suspicious locoregional nodal disease, rather than specifying the N classification according to the number of nodal involvement into N0, N1a, N1b, N1c, or N2 [39]. Even though the ESGAR group has tried to subclassify the clinical nodal status, its sensitivity remains unreliable. Moreover, due to the limitation of imaging performances, the differentiation between tumor deposits (TDs) and pathological lymph nodes remains challenging [40].
- MRF invasion: MRF+ is suspicious, if the tumor is located ≤1 mm from the mesorectal fascia. To determine the MRF status, a radiologist measures the shortest distance between the MRF and the outermost part of the tumor, including EMVI, tumor deposits (TDs), or any suspicious lymph nodes with disrupted capsule. Lymph nodes with an intact capsule are not considered involved as they are not associated with increased local recurrence rates. Since the MRF covers the surface of the levator ani inferiorly, low rectal tumors contacting or situated within 1 mm of the MRF over the surface of the levator ani are also regarded as the MRF+ tumors.
- Tumor deposits (TDs): According to the consensus from the international panel of pathologists have agreed that TDs have more adverse prognostic implications than lymph node metastases, and staged them as substage N1c is suboptimal as it does not adequately represent this risk status [41]. Intriguingly, baseline MRI-detected TDs (mrTD)/MRI-detected EMVI (mrEMVI) status was rather the only independent clinical factor determining clinical outcomes (HR 2.36 for OS, 2.37 for DFS (both with *p* < 0.001), 6.53 for DM (*p* ≤ 0.001), prevailing both clinical T and N categories. Those who had received neoadjuvant therapy, the post-treatment mrTD/mrEMVI status was also the only significant prognostic factor [42].
- The presence of mucin: Mucin-containing rectal cancer usually display high signal intensity on T2-weighted MRI. The mucinous tumor is associated with presentation at a younger age [42,43] and at higher a higher stage at the time of diagnosis with substantial more metastatic tendency [44].
- EMVI is defined as the presence of tumor cells within the blood vessels, beyond the muscularis propria and in the proximity of the rectal tumor; therefore, the tumor must be at least cT3 category Once EMVI+ is demonstrated, it is obligatory to evaluate the distance to the MRF since it is determined infiltrated (MRF+) when an EMVI is very close (≤1 mm) to the MRF.

According to the updated results from the RAPIDO trial disclosed that the post-TNT predictive factors associated with higher chance of LRR were enlarged lateral lymph nodes, CRM+, TDs, and pN+. This finding arouses us how to tailor the specifically intensive treatment strategy for such the very-high-risk patients would be a provocative research question. Besides the standard of care like TME, the optimal management of the lateral pelvic lymph nodes (LPLN) remains another debatable issue. Historically, two distinct paradigms in management of LARC with LPLN have developed in the East and West. In Japan, TME with LPLN dissection (LPLND) is the routine operation for locally advanced low rectal cancer [45]. On the contrary, a historical study from the West regarding the LPLND revealed unacceptable morbidities without meaningful clinical benefits [46]. Furthermore, the AJCC TNM classification ubiquitously used around the world actually categorizes an external iliac or obturator nodal metastasis as distant metastasis, thereby considering the LPLN involvement as a systemic disease. The addition of pre-operative (chemo)RT with or without the consolidation combination chemotherapy has become the more popular paradigm. A systematic review exploring functional outcomes after LPLND for rectal cancer demonstrated that TME with LPLND was related to worse male sexual dysfunction compared to TME only surgery (RR 1.68, 95% CI: 1.41–1.99) but found no significant difference in urinary dysfunction frequently reported by the cohort studies before the year 2000 [47]. When the nerve-sparing LPLND has been more and more routinely practiced, it would result in remarkably less genitourinary AEs reported by the more recent cohort studies compared with the earlier ones. Since the updated analysis from the RAPIDO trial demonstrated the impreciseness of post-neoadjuvant treatment response evaluation. The pre-operative TNT with TME and LPLND should be offered for those with highly suggestive of LPLN involvement based on the findings from pre-treatment high resolution pelvic MRI.

In conclusion, those with one of the following adverse features: cT4, cN+, MRF+, mrTD/mrEMVI deserve TNT. Those with cT3 (especially, cT3a/b) but without other pre-treatment adverse features can proceed to upfront TME. Adjuvant chemoradiotherapy with or without adjuvant chemotherapy is selectively considered, if surgical pathological report eventually reveals unexpected adverse features. For those with LPLN involvement, TNT is strongly recommended. TME with additional LPLND performed by an expert surgeon is solidly recommended.

## 7. Can We Omit Pre-Operative RT in Patients with LARC?

The concept of omission of pre-operative RT has been a subject to be challenged since the gathered results from clinical trials conducted in mainland China demonstrated equivalent outcomes at least in some cases with low-risk features. The FOWARC trial recruited 495 rectal (distal tumor border situated less than 12 cm from AV) cancer patients with clinical stage II and III with cN+ and randomly assigned into three arms of pre-operative treatments, LC-CRT (46–50.4 Gy/23–25 Fr) with concomitant LV5FU2 infusion followed by TME and adjuvant LV5FU2 for 7 cycles, LC-CRT with concomitant mFOLFOX6 and mFOLFOX6 alone (without RT) for 4–6 cycles followed by TME and adjuvant mFOLFOX6 for 6–8 cycles. Approximately one-third of the participants had cT4, and one-fourth of them had cN2 and one-third of them had MRF+. After a median follow-up of 45.2 months, no difference in 3-year DFS (the primary end point), LRR rates and OS was reported. 3-year DFS was 72.9%, 77.2%, and 73.5% (*p* = 0.709), the 3-year LRR rate (after R0/1 resection) was 8.0%, 7.0%, and 8.3% (*p* = 0.873), and the 3-year OS rate was 91.3%, 89.1%, and 90.7% (*p* = 0.971), respectively. Presence of TDs and pathological residual diseases (ypTNM stage II/III and poor tumor regression rate (2–3 vs. 0–1)) were the independent predictive factors of inferior DFS. Provocatively, pre-operative mFOLFOX6 only was significantly related to intact bowel function [48]. The FORWARC study has many strengths especially the fact that the investigators used prolonged infusion of 5-FU concomitant with RT, supposed to be superior to bolus 5-FU administration during RT [49,50]. This study also supported the earlier trials that the addition of oxaliplatin to 5-FU- or capecitabine-based chemotherapy in concomitant with pre-operative RT had failed to demonstrate the benefit in the improved response rate [49], LRR rate and DFS [51,52]. As expected, oxaliplatin in concomitant with RT significantly increased toxicities. However, the FOWARC study was not designed with enough power to detect either non-inferiority or superiority of omission of pre-operative RT. The CONVERT trial, also conducted in China was another phase 3 study specifically designed to compare neoadjuvant CAPOX only (nCT) with neoadjuvant LC-CRT with concomitant capecitabine (nCRT). There were 589 locally advanced cT2N+ or cT3/4aNany rectal (within 12 cm from AV) cancer patients without MRF involvement (MRF-) as determined by pelvic MRI enrolled and randomized 1:1 into either nCT or nCRT. TME was planned to be performed within 2 to 4 weeks after nCT and 6 to 10 weeks after nCRT. Regardless of pathologic responses, patients in the nCT group received another 4 cycles of CAPOX, and patients in the nCRT group received 6 cycles of adjuvant CAPOX. Approximately 30% of participants had cN0, 80% had mEMVI- (MRI-defined negative extramural vascular invasion) and 90% had mLPLN-. The primary end point is LRR-free survival. Reports from early data analysis revealed no difference in the pCR rate (11.0% vs. 13.8%; *p* = 0.33) as well as the downstaging (yp stage 0 to 1) rate (40.8% vs. 45.6%; *p* = 0.27). Curiously, nCT was significantly related to lower peri-operative distant metastases rate (0.7% vs. 3.1%; *p* = 0.03) and preventive ileostomy rate (52.2% vs. 63.6%; *p* = 0.008) compared with nCRT [53]. However, more relevant outcomes like the LRR rate and DFS were still pending. The latest ground-breaking trial, the phase 2/3 non-inferiority PROSPECT trial was a study particularly designed to demonstrate the feasibility of omission of pre-operative RT. There were 1194 participants with cT2N+ and cT3N+/− (excluding cT4 tumors and ≥ 4 pelvic lymph nodes with a short axis larger than 10 mm) and MRF- (as defined as tumor visible not closer than 3 mm from the radial margin) based on the baseline MRI imaging enrolled and randomized 1:1 into the nCT group (585 patients, 6 cycles of mFOLFOX6) and the LC-CRT group (543 patients, 50.4 Gy/28 Fr concomitant with infusional 5-FU or capecitabine). Adjuvant FOLFOX was optional. The protocol indicated that the participants in the nCT arm who could not complete at least 5 cycles of FOLFOX or had tumor regression of less than 20% as determined by the surgeon on the basis of restaging imaging, proctoscopy, and physical examination had to receive pre-operative CRT, instead. Post-operative CRT was recommended for patients in the nCT group who did not acquire R0 resection. After a median follow-up of 58 months, the non-inferiority of neoadjuvant FOLFOX only was proved in terms of DFS (HR 0.92; 90.2% CI, 0.74–1.14; *p* = 0.005 for non-inferiority). 5-year DFS was 80.8% vs. 78.6% in the nCT and LC-CRT, respectively. Both the OS (HR 1.04; 95% CI, 0.74–1.44) and the LRR rate (HR 1.18; 95% CI, 0.44–3.16) were also similar. Notably, 53 patients (9.1%) required pre-operative CRT and 8 (1.4%) eventually received post-operative CRT [8]. Worth to be mentioned here is the fact that the PROSPECT enrolled very-low-risk patients with high tumor location (approximately one-fifth of the participants had tumor 10 cm above the anal verge) and patients with cT3N0 (approximately one-third had cT3N0) who could safely undergo upfront operation based on the widely accepted current standard of care. Most of the participants (approximately two-third) had tumor located in mid rectum (5–10 cm from AV). Whether sparing RT in patients with low-lying (0–5 cm from AV) cT3N+ rectal cancer is a worrisome issue. Furthermore, the criteria of poor response to nCT assessed pre-operatively was too subjective and its necessity was also debatable since the post-operative CRT was the possible alternative. 

Gao et al. retrospectively reviewed the data from stage II/III rectal cancer patients who underwent curative surgery without pre-operative CRT. Those with upper rectal cancer defined as lesions above the APR as determined by MRI had significantly lower chances of LRR than those straddle or below the anterior peritoneal reflection (APR) (*p* = 0.042). No significant difference in LRR whether pre-operative RT was applied among them. The tumor location regarding APR was an independent predictor of adverse LRR-free survival [54]. 

Unanimously, the most provocative breakthrough in management of LARC is the offering neoadjuvant immune-checkpoint blockade for patients harboring mismatch repair–deficient (dMMR)/microsatellite instability-high (MSI-H). Since patients with dMMR/MSI-H metastatic colorectal adenocarcinoma respond to anti-programmed death 1 (PD-1) substantially and enduringly compared with conventional chemotherapy as demonstrated by the KEYNOTE-177 trial using pembrolizumab [55]. Therefore, it was hypothesized that an immune-checkpoint blockade could be effective in downsizing rectal tumor with dMMR as well. Cercek et al. conducted a pioneered phase 2 study in which single-agent dostarlimab, an anti–PD-1, was administered every 3 weeks for 6 months in 12 patients with dMMR (loss of expression of one of the following MMR proteins, MLH1, MSH1, MSH6, and PMS2) stage II or III rectal cancer. Those who achieved a cCR after completion of dostarlimab therapy were allowed to proceed to the watch-and-wait strategy (without CRT and surgery). Astoundingly, cCR was accomplished in all of the 12 participants who were enrolled for longer than 6 months, without evidence of tumor on any means (MRI, PET, endoscopic evaluation, digital rectal examination, or biopsy). The study reported an interim analysis after median follow-up of 12 months that no patients required salvage CRT or underwent surgery. Not even a case developed progressive disease or recurrence during follow-up (range, 6–25 months). In addition, no serious adverse events was detected [56]. The USFDA has immediately granted accelerated approval for dostarlimab for patients with mismatch-repair-deficient (dMMR) metastatic cancer. At this moment, we still lack of data regarding the durability of response. 

In conclusion, patients with cT1–2N0 and cT3a/bN0 with MRF- (as determined by high-resolution pelvic MRI) or upper rectal tumor (more properly called as the rectosigmoid carcinoma) can safely proceed to upfront surgery. Those with intermediate-risk features as defined by the PROSPECT trial, i.e., cT2N+ and cT3N+/− (not more than 3 pelvic lymph nodes with a short axis larger than 10 mm) and MRF- (tumor visible not closer than 3 mm from the radial margin) are the candidates for neoadjuvant mFOLFOX6 for 6 cycles without pre-operative RT prior to surgery; however, those who fail to response appreciably to pre-operative chemotherapy should receive conventional LC-CRT. Additional adjuvant chemotherapy is under a clinician’s discretion. In addition, the watch-and-wait protocol is an option for low- to intermediate-risk LARC who achieve cCR after nCT or neoadjuvant SC-RT or LC-RT.

## 8. Is Adjuvant Chemotherapy (AC) Necessary after Completion of TNT and TME?

Patients with LARC were particularly banned from the landmark adjuvant chemotherapy (AC) studies due to the confounding impact of prior (chemo)radiotherapy on treatment effects. Historically, the adjuvant chemotherapy compliance was usually poorer, if it was administered to patients who had ever received intensified pre-operative therapy. Most of the TNT trials did not mandate the adjuvant post-operative chemotherapy. Hence, the contributary effect of AC on survival after receiving TNT was impossibly estimated. Clinicians usually extrapolate the benefits of AC for patients with LARC from the pivotal adjuvant colon cancer studies (MOSAIC and NSABP C-07) [57,58]. Even though various studies have endeavored to elucidate the benefits of AC following the standard-of-care pre-operative (chemo)radiotherapy and curative resection, the results are incongruous. A meta-analysis by Breugom et al. based on data from 1196 patients who received fluoropyrimidine-based AC (5-FU/LV, capecitabine or CAPOX) after pre-operative (chemo)radiotherapy and surgery unfolded the unexpectedly insignificant improvement in OS between patients who received AC and those who did not (HR 0.97; 95% CI, 0.81–1.17; *p* = 0.775). Furthermore, no specific subgroup that gained OS benefit from AC was found. Although, in subgroup analyses, patients with higher lesions (10–15 cm from the anal verge) tended to acquire DFS benefit (HR 0.59; *p* = 0.005, *p* (interaction) = 0.107) and fewer distant recurrences (HR 0.61; *p* = 0.025, *p* (interaction) = 0.126), AC failed to demonstrate significant improvement DFS among overall participants (HR 0.91, 95% CI 0.77–1.07; *p* = 0.230) or distant recurrences (0.94, 0.78–1.14; *p* = 0.523) compared with observation [59]. The PETACC-06 study recruited patients who had undergone pre-operative LC-CRT with capecitabine prior to surgery and compared DFS between those who received adjuvant capecitabine for 6 cycles and those who received CAPOX for equal number of cycles. The updated 7-year DFS (66.1% vs. 65.5%; HR 1.02) and OS (73.5% vs. 73.7%; HR 1.19) rates revealed insignificant benefits of adding oxaliplatin [60]. The final analysis of CAO/ARO/AIO-04 study comparing pre-operative LC-CRT with infusional 5-FU followed by surgery and adjuvant bolus 5-FU/LV for 4 cycles with pre-operative LC-CRT with infusional 5-FU and bolus oxaliplatin followed by surgery and adjuvant FOLFOX for 8 cycles disclosed a slight improvement in 3-year DFS of adding oxaliplatin (75.9% vs. 71.2%; HR 0.79; 95% CI, 0.64–0.98; *p* = 0.03) [61]. In accordance with the CAO/ARO/AIO-04 study, the long-term results from the ADORE study comparing adjuvant 5FU/LV with FOLFOX among patients who had undergone pre-operative LC-CRT prior to surgery revealed that adjuvant FOLFOX improved 6-year DFS rate (68.2% vs. 56.8%; HR 0.63; 95% CI, 0.43–0.93; *p* = 0.018). The subgroup analysis for DFS also demonstrated clinical benefit of FOLFOX over 5-FU/LV in patients with residual tumor after pre-operative therapy (yp stage III, ypN1b, ypN2, minimally regressed tumor), high-grade histology, and an absence of lymphovascular or perineural invasion. Nevertheless, FOLFOX failed to boost the 6-year OS rate (78.1% vs. 76.4%; HR 0.73; 95% CI, 0.45–1.19; *p* = 0.21). Adjuvant FOLFOX seemed to be more favorable option among those with ypN2 and minimally regressed tumor [62]. Arguably, both of the previously mentioned trials were not specifically designed to compete with observation only. The OS benefit of AC among those who receive pre-operative (chemo)RT has still been mysterious.

In conclusion, although AC after completion of TNT and TME is still debatable, the author suggests adjuvant FOLFOX/CAPOX for patients with LARC who harbor high-risk features of developing distant metastasis, i.e., cN+ and poorly regressed tumor. Provided that such patients can still tolerate its adverse effects of oxaliplatin, especially the neurological toxicities, cumulative cycles of oxaliplatin-based chemotherapy administered in both pre-operative and post-operative settings should not exceed 8 cycles of CAPOX and 12 cycles of FOLFOX.

## 9. How Do We Treat the Elderly?

Elderly cancer patients are usually underrepresented in clinical trials, even though the majority of cancer patients age 65 years or more. If data specifically explored in the elderly cancer patients are available, most of them showed the marginal or insignificant survival benefits. The pooled analysis of adjuvant oxaliplatin-based chemotherapy in patients with colorectal cancer aged more than 70 years also failed to demonstrate OS benefit (HR in the elderly subgroup, 1.02; 95% CI, 0.82–1.27 vs. HR in the non-elderly subgroup, 0.84; 95% CI, 0.72–0.97) [63]. Regarding the tolerability of neoadjuvant treatment, Hamed et al. conducted a scoping review of the clinical data from 33 studies published during 2005 to 2022 (most of them were the retrospective studies) and disclosed that among elderly rectal cancer patients, LC-CRT led to more frequent toxicities and treatment interruptions. Convincingly, SC-RT was rather more tolerable. SC-RT also had a tendency towards better survival [64]. The mature data from the PRODIGE 42-GERIO 12 NACREE trial specifically designed to compare pre-operative LC-CRT with delayed surgery and SC-RT with delayed surgery among elderly patients with cT3/4 rectal cancer aged more than 75 years old would provide us the definite conclusion. A multidisciplinary approach incorporating multidimensional geriatric assessment (GA) prior to making a specific therapeutic decision is indispensable to select the safest management and ameliorate the treatment-related toxicities.

In conclusion, the multidimensional geriatric assessment should be performed prior to commencement of therapy. In fit elderly patients, pre-operative SC-RT or even TNT is a possible option. In pre-frail elderly patients, pre-operative SC-RT is a more suitable option. On the contrary, in frail elderly and “symptomatic” patients, palliative therapy with SC-RT and/or discharge colostomy would be a safer option.

## 10. Conclusions and Recommendation

Until the mature data from the ACO/ARO/AIO-18.1 [65] randomized trial are revealed, whether SC-RT or LC-CRT followed by the consolidation chemotherapy and TME should be the best option for patients with intermediate-to-high-risk LARC (one of the following features (1) any cT3, if low rectal (0–6 cm from anal verge); (2) cT3c/d mid rectal (6–12 cm from AV); (3) cT4; (4) anyT with cN+; (5) mMRF+ (≤1 mm); (6) EMVI+) is still inconclusive. To select the most proper management, the author suggests that a physician should consider not only the tumor biological aspect (tumor stage, tumor aggressiveness and response to neoadjuvant or TNT therapy) but also the patient’s condition (age, physical fitness and co-morbidities), and acceptance of the treatment consequences, especially adverse events caused by the multimodality therapy. Table 2 showed the recommendation for LARC based on the recent mature clinical data. 

## Figures and Tables

**Table 2:** Showed the recommendation of the most appropriate management paradigm for patients with locally advanced rectal cancer.

Risk Stratification (Based on High-Resolution Pelvic MRI)	Definition	Suggested Management
Low risk	cT2N0, High rectal (above 12 cm from AV) cT3a/b * N0, tumors above the sigmoid take-off without peritoneal invasion	- Upfront surgery
Intermediate	Mid-rectal cT3a/b * N0 or anyT with N+ and MRF- (≤3 mm **)	- mFOLFOX6 × 6 cycles → TME (WWP is another option for those achieve cCR) (Consider pre-operative LC-CRT, if primary tumor decreases in size less than 20% as determined by physical examination, pelvic imaging and rectal endoscopy. Post-operative LC-CRT is recommended if resection is not pathologically complete (R0)) ** - SC-RT with delayed surgery (9–12 weeks) → TME (WWP is another option for those achieve cCR) (Consider adjuvant FOLFOX/CAPOX, even though there are scant evidences supporting its survival benefits)
High	Low rectal (0–5 cm from AV)cT3; mid-rectal (5–10 cm from AV) cT3c/d *; cT4; cN+; MRF+ (≤1 mm); EMVI+; TDs+	Either SC-RT or LC-CRT followed by consolidation chemotherapy → TME (Consider adjuvant FOLFOX/CAPOX in patients with poorly regressed tumor, even though there are scant evidences supporting its survival benefits)
Very high	Multiple lateral pelvic lymph nodes (LPLNs)	Either SC-RT or LC-CRT followed by consolidation chemotherapy → TME with LPLND and adjuvant FOLFOX/CAPOX ***

* cT3a/b: <5mm extramural invasion; cT3c/d: ≥5 mm extramural invasion. ** According to the PROSPECT trial, most (approximately 2/3) of the participants had mid-rectal tumors; therefore, the author does not advocate neoadjuvant chemotherapy only for low-lying cT3 tumors. *** According to the updated data from the RAPIDO trial, the author suggests that the determination of extent of surgical resection (i.e., sphincter preservation or permanent colostomy) should be based on pre-treatment staging evaluations rather than post-treatment ones. The watch-and-wait strategy is discouraged. Furthermore, the nerve-sparing lateral pelvic lymph node dissection is strongly recommended in any patients with suspicious lateral pelvic lymph nodes regardless of presence of other high-risk features.
